# Nutritional Factors Affecting Uremic Toxin Production

**DOI:** 10.3390/toxins18050223

**Published:** 2026-05-08

**Authors:** Fanny Jouve, Christophe O. Soulage, Laetitia Koppe

**Affiliations:** 1 CarMeN, INSERM U1060, INRA U1397, Claude Bernard Lyon 1 Université, 69310 Pierre-Bénite, France; fanny.jouve@univ-lyon1.fr (F.J.); christophe.soulage@univ-lyon1.fr (C.O.S.); 2 Department of Nephrology and Nutrition, Hospices Civils de Lyon, Centre Hospitalier Lyon-Sud, 69310 Pierre-Bénite, France; 3 Lyon GEM Microbiota Study Group, 69000 Lyon, France

**Keywords:** chronic kidney disease, nutrition, uremic toxins, gut microbiota

## Abstract

Chronic kidney disease (CKD) is characterized by persistent exposure to uremic toxins (UTs), many of which originate from gut microbial metabolism and contribute to renal, cardiovascular, and metabolic complications. Current evidence indicates that CKD is associated with dysbiosis and the enrichment of microbial taxa carrying genes involved in UT precursor production. Diet is a major modulator of the gut microbiota and therefore represents a promising lever to reduce UT generation in synergy with current nephroprotective therapies. Beyond simple protein restriction, more specific dietary approaches, particularly plant-based low-protein diets, appear especially relevant. Additional factors, including amino acid composition, lipid quality, food processing, constipation, transit time, meal timing, and circadian rhythms, may also influence microbial metabolism and UT production. This review examines the role of nutrition in shaping the gut microbiota–UT–kidney axis and discusses how dietary modulation may support precision nutrition in the context of CKD. It also highlights future directions based on multidimensional phenotyping and robust biomarkers to capture interindividual variability, guide personalized interventions, and ultimately improve renal and cardiovascular outcomes in CKD.

## 1. Introduction

Nutrition remains a cornerstone in the management of chronic kidney disease (CKD), acting in synergy with recent pharmacological advances such as sodium–glucose cotransporter 2 inhibitors (SGLT2is), renin–angiotensin–aldosterone system inhibitors (RAASis), and glucagon-like peptide-1 receptor agonists (GLP-1RAs). Beyond sharing common risk factors with metabolic disorders including diabetes, obesity, metabolic dysfunction-associated steatotic liver disease (MASLD), and cardiovascular (CV) diseases, CKD is characterized by chronic and prolonged exposure to bioactive metabolites known as uremic toxins (UTs), which exert many deleterious systemic effects.

A major challenge in CKD management is that, aside from dialysis, effective strategies to eliminate these toxins remain limited, particularly for protein-bound UTs that are poorly cleared by most conventional dialysis techniques. Recent experimental and clinical data have nevertheless improved the classification of UTs according to their origin and biological activity, allowing for better identification of actionable sources. Notably, a large proportion of these metabolites originate from the intestinal microbiota. Therefore, the accumulation and high concentrations of UTs in the bloodstream of CKD patients result from a combination of impaired kidney function leading to reduced clearance, increased de novo production driven by CKD-associated dysbiosis, and enhanced gut permeability (often referred to as “leaky gut”) [1]. Indeed, several studies have underlined that both gut microbiota composition and function are markedly altered in CKD, with enrichment of bacteria carrying enzymes involved in UT production [1,2,3]. Human lifestyle, particularly in Western societies, together with environmental factors related to diet such as ambient temperature and circadian rhythms [4,5] have also emerged as important and previously underrecognized determinants of microbiota structure and function.

In this context, a deeper understanding of the gut–kidney axis provides new opportunities to modulate the gut microbiota to reduce the production and absorption of UTs and ameliorate CKD progression and outcomes. A healthy diet represents one of the most powerful modulators of gut microbiota composition and function, shaping microbial metabolic activity and host–microbe interactions with profound systemic consequences.

However, several critical questions remain unanswered, including the identification of optimal dietary patterns to limit UT generation, the feasibility of personalized nutritional interventions, and the importance of dietary composition beyond macronutrient intake. Emerging evidence also suggests that meal timing and dietary rhythms may play an important role in CKD progression. Advances in biomarker-driven approaches offer promising tools to refine nutritional strategies by capturing interindividual variability and enabling precision nutrition in CKD aimed at reducing UT accumulation.

The aim of this review is to discuss the importance of diet as a key driver of host–microbe symbiosis and gut microbe-derived bioactive metabolite production, provide an overview of current knowledge on the impact of nutrition on gut microbiota composition and function, and focus specifically on dietary modulation of UT production. Although many UTs are derived from the gut microbiota, this review focuses on the most studied metabolites with the strongest evidence: indole derivatives [indoxyl sulfate (IS) and indole-3-acetic acid (IAA)], phenolic compounds [p-cresyl sulfate (PCS) and p-cresyl glucuronide (PCG)], and TMAO (trimethylamine N-oxide).

## 2. Gut Microbiota Characterization as a Prerequisite for Personalized Nutrition in CKD

Knowledge of an individual’s gut microbiota may help optimize dietary interventions, as customization appears necessary for maximal benefit. Microbiota responses vary largely between subjects, highlighting strong interindividual variability. Studies of responders to targeted dietary interventions have provided insights into microbial ecology in diabetes for example [6]. Stratification based on baseline microbiota composition, metabolite profiles, microbial richness, and dietary response has been proposed, although most studies involved obese or otherwise healthy individuals [7]. The CKD context further increases complexity due to disease-related metabolic alterations and several treatment-related confounders.

Dietary approaches, including nutritional supplements such as probiotics or prebiotics, to modulate the microbiota and thereby reduce UT production in CKD largely derive from strategies developed to target other metabolic diseases [8]. However, not all these dietary approaches can be directly transposed to CKD. For example, probiotics that may be beneficial in metabolic diseases could be detrimental in CKD, as they may contain urease-producing bacteria that exacerbate ammonia generation from urea, while the uremic gut environment may also limit probiotic survival and benefits [8]. To better understand diet–microbiota interactions in CKD, characterization of the baseline gut microbiota under uremic conditions is pivotal. In this context, microbiota characterization should not be limited to simple taxonomic profiling or 16S rRNA gene sequencing but should rely on more informative approaches such as shotgun metagenomics, which enables both taxonomic resolution and functional profiling of bacterial communities. Additional parameters, including intestinal permeability and transit time, are also important to consider. Finally, complementary approaches, such as in vitro experiments, are essential to directly assess the production of UTs (e.g., indole, p-cresol, or trimethylamine), as post-transcriptional and metabolic regulation may influence bacterial function beyond what is captured by genomic analyses alone. Therefore, recent methodological recommendations specific to CKD research aim to improve study quality and comparability across cohorts [1]. Accordingly, several large-scale studies across CKD stages have integrated metagenomics with detailed clinical covariates, including diet and medications (as summarized in Table 1), consistently reporting the enrichment of taxa harboring genes involved in UT production, in particular tryptophanase and tyrosinase. However, most studies remain descriptive with limited functional insight. It is important to carefully account for confounders when analyzing the gut microbiota. Among the host covariates evaluated in relation to CKD and microbiota composition, diet was not the most influential, whereas medications were by far the most important [3]. Therefore, to accurately assess the specific impact of diet, these confounding factors must be carefully considered. Although artificial gut models offer promise for studying the impact of diet and nutrition on UT production, they remain technically challenging, costly, and difficult to scale [9].

However, our current exploration of the gut microbiome remains partial. First, few studies have extended beyond the bacteriome to evaluate the roles of the virome, mycobiome, and archaeome, which also constitute key components of the human gut ecosystem [10]. Second, investigations of microbiota-derived metabolites have largely focused on a limited number of compounds, often prioritizing toxic by-products such as trimethylamine, p-cresol, and indole (respectively the precursors of trimethylamine N-oxide—TMAO, p-cresyl sulfate—PCS, and indoxyl sulfate—IS), while neglecting beneficial metabolites beyond short-chain fatty acids (SCFAs) and, importantly, failing to assess the balance and interplay between harmful and protective microbial by-products. Nevertheless, advances in high-throughput omics and sequencing technologies are expected to enable more comprehensive characterization of these components in the future [11,12].

Finally, the accumulation of UTs within the gut, such as urea and IS, may contribute to dysbiosis. For example, urea may increase intestinal permeability [13], while IS may promote the growth of bacteria such as Enterobacteriaceae [14]. This, in turn, may enhance the production and accumulation of uremic solutes, including indole and urea, thereby exacerbating kidney injury and further increasing their levels. This illustrates the bidirectional nature of the gut–kidney axis.

**Table 1:** Principal studies (including >100 patients) exploring the gut microbiota using a metagenomic approach in patients with CKD.

Study	Population	Main Results
Lin et al., 2026 [15]	General population, n = 494	Gut microbial diversity and 44 reproducible species are independently linked to kidney function.
Laiola et al., 2025 [2]	CKD stage 3–5, n = 240	Patients with severe CKD show greater enrichment of UT precursor-producing species in the microbiota than patients with moderate CKD.
Krukowski et al., 2025 [3]	CKD stage 1–5, n = 130	Lower eGFR was associated with increased p-cresol and indole biosynthetic potential and reduced plant-to-animal CAZyme ratios.
Hellman et al., 2025 [16]	General population n > 7000	Decreased gut microbial diversity may be associated with the risk of CKD.
Lin et al., 2025 [17]	General population, n= 1555	*Segatella copri* was positively associated with kidney function through microbial ammonia metabolism-related pathways and the asnA gene, which encodes an ammonia-assimilating enzyme.
Zhang et al., 2025 [18]	HD, n = 255, and CKD stage 3–5, n = 171	Numerous viral functional signatures were associated with CKD, notably a marked reduction in nicotinamide adenine dinucleotide synthesis capacity.
Cheng et al., 2025 [19]	CKD stage 3–5, n = 152	Rapid eGFR decline was associated with a distinct gut microbial composition characterized by increased α-diversity and an abundance of TMA-producing bacteria.
Wang et al., 2020 [20]	HD, n = 223	CKD is associated with bacterial-encoded functions involved in toxin and secondary bile acid synthesis.

Abbreviation: asnA: asparagine synthase A gene encoding an ammonia-assimilating enzyme; CAZyme: carbohydrate-active enzyme; CKD: chronic kidney disease; eGFR: estimated glomerular filtration rate; HD: hemodialysis; TMA: trimethylamine; UT: uremic toxin.

## 3. Effects of Specific Nutrients on the Gut Microbiome and Uremic Toxin Production in CKD

Traditional dietary advice for CKD has mainly focused on energy and protein intake and on restricting minerals such as potassium, phosphorus and calcium [21]. The strongest evidence, reflected in the KDOQI guidelines, supports low-protein diets (LPDs; 0.55–0.60 g/kg/day) or very-low-protein diets (VLPDs; 0.28–0.43 g/kg/day) while maintaining sufficient energy intake (~25 kcal/kg/day) [22]. These approaches are associated with improved outcomes, including delayed CKD progression and a lower risk of protein–energy wasting [21]. However, a better understanding of individual dietary substrates and their combinations influencing UT production is essential to advance personalized nutrition and its impact on CKD progression and complications [10,23,24].

In Table 2, we summarize the main dietary intervention studies evaluating UT concentrations in adult patients with CKD. However, several important limitations should be considered. First, most studies have small sample sizes, short follow-up durations, and heterogeneous patient populations, including different CKD stages, dialysis modalities, and comorbidities. These factors contribute to variability in outcomes and limit generalizability. Second, there is substantial heterogeneity in the methods used to quantify UTs. Differences between total and free (protein-bound) fractions, as well as between targeted assays and untargeted metabolomics approaches, can lead to significant discrepancies in reported concentrations and reduce comparability across studies. Pre-analytical factors, such as fasting versus fed state, may also influence measured levels. In addition, variability in microbiota assessment methods, lack of standardized protocols, and generally small effect sizes further contribute to inconsistent findings. Altogether, these limitations highlight the need for larger, well-designed studies with standardized methodologies, including harmonized UT measurements and microbiota profiling, as well as longer follow-up and more homogeneous populations, to better define the impact of dietary interventions in CKD.

### 3.1. Proteins

#### 3.1.1. Low-Protein Diets

LPDs were initially recommended to reduce kidney hyperfiltration. Beyond their hemodynamic effects, high protein intake (>1.3 g/kg/day) promotes the expansion of proteolytic bacteria (e.g., *Bacteroides* and *Enterobacteriaceae*) [25], thereby enhancing gut-derived UT production such as IS and PCS. Conversely, LPDs appear first to reduce the precursors of several UTs, including tryptophan, tyrosine, and carnitine (respectively precursors of p-cresol, indole and TMA), but also to induce favorable shifts in the gut microbiota towards butyrate-producing and anti-inflammatory taxa while reducing dysbiosis, as summarized in Table 2. In CKD, emerging observational data indicate a progressive shift from saccharolytic to proteolytic metabolism with declining kidney function [3]. In vitro systems colonized with fecal samples from patients with CKD also show increased basal production of p-cresol and indole, particularly when the system is fed with protein, along with the presence of branched-chain fatty acids, reflecting enhanced protein fermentation and providing an additional argument to reduce protein intake [9]. However, evidence remains limited, with small patient numbers and short exposure periods. Reported microbiota changes occur mainly at the family and species levels, with minimal effects on diversity or richness and insufficient metabolic or clinical impact, as highlighted in a recent systematic review [26]. This meta-analysis relied on 16S rRNA gene sequencing, which provides limited functional insight. In addition, among the studies analyzed, only a subset assessed UTs, and these were limited to plasma measurements, which are difficult to interpret as they reflect both production and accumulation and are strongly influenced by renal function. Assessments of feces would be more informative for production, whereas urinary measurements may better reflect excretion. This may partly explain the absence of a clear association. The use of AST-120, an intestinal adsorbent that is highly effective in reducing UTs in animal models, has not demonstrated efficacy on renal progression in large clinical trials. However, in these studies, key UTs such as PCS and IS were not measured [27]. This raises the question of whether the lack of clinical benefit may be related to an insufficient reduction in UT levels. Altogether, this underscores the need for larger exploratory studies to determine whether modulation of the gut microbiota and UT production could translate into meaningful clinical improvement.

**Table 2:** The effects of the main dietary interventions on uremic toxin concentrations in adult patients with CKD (limited to the last 10 years).

Study	Diet	Time	Population	Gut Microbiota Analysis	Main Finding
Low-protein diet					
Koppe et al., 2026 [28]	RCT, FD (0.8 g/kg/day) vs. VLPD (0.4 g/kg/day + KAs)	3 months	CKD stage 4–5; n = 18	Yes	↓ serum IS, PCS, TMAO ↓ bacteria carrying UT genes
De Mauri et al., 2022 [29]	Cross-sectional study, adherence to LPD (0.6 g/kg/day)	NA	CKD stage 4–5; n = 87	No	↓ serum IS and PCS
Chang et al., 2023 [30]	RCT, LPD (0.6–0.8 g/kg/day) vs. LPD + KAs).	6 months	CKD stage 3b-4; n = 22	No	↓ serum IS and PCS
Black et al., 2018 [31]	Observational, adherence to LPD (0.6 g/kg/day)	NA	CKD stage 3–4; n = 30	No	↓ serum PCS
Plant-based diet and fiber supplementation					
Headley et al., 2025 [32]	RCT, amylose-RS	16 wks	CKD stage 3B-4; n = 68	Yes	No changes in microbial beta diversity ↑ microbial α-diversity ↓ serum PCS
Standfort et al., 2025 [33]	Crossover RCT, high-diversity plant-based diet (≥30 unique plant foods weekly) vs. low-diversity plant-based diet (≤15 unique plant foods weekly)	4 wks	CKD stage 3–4, n = 25	Yes	↓ urinary and serum UTs in patients with poorer kidney function and higher baseline UT levels Shift in the gut microbiome toward increased production of beneficial metabolites such as butyrate and isobutyrate ↑ microbial diversity
Pajk et al., 2025 [34]	RCT, MD	4 wks	PD; n = 21	No	No difference in serum IS and PCS
Chang et al., 2023 [35]	RCT, LPD (0.6–0.8 g/kg/day) vs. LPD (0.6–0.8 g/kg/day) + 10 g/d of wheat starch/day	12 wks	CKD stage 3b-5, n = 45	No	↓ serum IS and PCS and inflammation index
Hansen et al., 2023 [36]	RCT, New Nordic Renal Diet	1 wk	CKD stage 3–5, n = 18	No	↓ urinary PCS and IS
Tsai et al., 2023 [37]	Crossover RCT, low phosphorus-to-protein ratio, higher portions of plant-based food, and high fiber content	1 wk	HD, n = 33	No	↓ serum IS
Ebersolt et al., 2022 [38]	Cross-sectional study, protein/fiber index	NA	HD, n = 58	No	No correlation between serum IS or PCS concentration and protein/fiber index
McFarlane et al., 2022 [39]	Cross-sectional study; plant-based diet index, protein intake, fiber intake, and dietary protein-to-fiber ratio	NA	CKD stage 3B-4; n = 68	Yes	↓ fibers and serum IS and PCS Healthy plant-based diet index was associated with lower levels of serum PCS Higher protein-to-fiber ratio ↑ *Oscillospirale*
Di Iorio et al., 2019 [40]	Crossover RCT, FD (0.8 g/kg/day) vs. VLPD (0.3 g/kg/day + KA) vs. MD	2 months	CKD stage 3B-4; n = 60	Yes	VLPD and MD: ↓ serum IS and PCS ↑ butyrate-forming species of *Lachnospiraceae, Ruminococcaceae, Prevotellaceae* VLPD: ↓ inflammatory *Proteobacteria* and ↑ *Actinobacteria* phyla
Ebrahim et al., 2022 [41]	RCT, 13.5 g/day of ß-glucan prebiotic fibers	14 wks	CKD stage 3B-4; n = 59	Yes	↓ serum PCS, IS, PCS No differences in abundances of genera
Ramos et al., 2019 [42]	RCT, prebiotic (FOS, 12 g/day)	3 months	CKD 3B-5, n = 50	No	↓ serum PCS
Esgalhado et al., 2018 [43]	RCT, RS (16 g/day)	4 wks	HD, n = 31	No	↓ serum IS and IL-6
Elamin et al., 2017 [44]	RCT, 10, 20, or 40 g of gum arabic/day	4 wks	CKD stage 3B-4; n = 36	No	No difference in serum IS
Poesen et al., 2016 [45]	RCT; 10 g/day arabinoxylan oligosaccharides	4 wks	CKD stage 3–5; n = 28	No	No difference in serum PCS and IS
Rossi et al., 2015 [46]	Cross-sectional study, protein–fiber index	NA	CKD stage 4–5; n = 40	No	Dietary protein–fiber index is associated with ↓ serum IS and PCS
Salmean et al., 2015 [47]	RCT; 10 g/day pea hull for the first four weeks, 25 g/day for following weeks	12 wks	CKD stage 3–5; n = 70	No	↓ serum PCS
Polyphenol supplementation					
Kemp et al., 2025 [48]	RCT, royal jelly + 400 mg green propolis/day	2 months	HD, n = 38	No	No difference in serum PCS and IS
Nascumento et al., 2025 [49]	RCT, 130 mg curcuminoids/day + 110 mg/day of green propolis + ginger + lemon juice	8 wks	HD, n = 35	No	Tendency ↓ serum IAA
Reis et al., 2024 [50]	RCT, 500 mg/day of curcumin	12 wks	PD, n = 48	No	Tendency ↓ serum PCS
Fonseca L et al., 2024 [51]	RCT, 400 mg of green propolis/day	8 wks	HD, n = 42	Yes	No difference in serum PCS and IS *Fusobacteria species* positive correlation with IS *Firmicutes, Lentisphaerae*, and *Proteobacteria* phyla negatively correlated with IS
Alvarenga et al., 2022 [52]	Crossover RCT, 500 mg of trans-resveratrol/day	4 wks	CKD 3B-5, n = 20	No	No difference in serum IS, pCS and IAA
Pivari et al., 2022 [53]	RCT, 500 mg of curcumin twice a day	6 months	CKD stage 3b-4 n = 24	Yes	↑*Lactobacillaceae spp.* ↓*Escherichia-Shigella* No difference in serum PCS and IS
Teixeira et al. 2022 [54]	RCT, 500 mg of dry cranberry extract twice a day	2 months	CKD stage 3B-4, n = 25	No	No difference in serum PCS and IS
Saralolli et al. 2021 [55]	RCT, 100 mL of orange juice with 12 g carrot and 2.5 g of turmeric/day	3 months	HD, n = 28	No	↓ serum PCS
de Andrade et al., 2021 [56]	RCT, 21 g/day unripe banana flour (RS)	4 wks	PD, n = 43	No	No difference in serum PCS and IS

Abbreviation: CKD: chronic kidney disease; FD: fiber diet; HD: hemodialysis; IAA: indole-3-acetic acid; IS: indoxyl sulfate; LPD: low-protein diet; LPD + KAs: low-protein diet supplemented with ketoanalogues; MD: Mediterranean diet; PCS: p-cresyl sulfate; PD: peritoneal dialysis; RCT: randomized controlled trial; RS: resistant starch; VLPD: very-low-protein diet; wks: weeks.

#### 3.1.2. Low-Protein Plant-Based Diets

Beyond protein quantity, dietary protein source is a major determinant of gut microbiota modulation. Plant- and animal-derived proteins differentially shape microbial communities, partly due to the presence of carbohydrates, fibers, and bioactive compounds such as polyphenols. Controlled human studies specifically addressing the effects of protein sources remain limited, with most evidence derived from animal models, the majority without CKD [57]. For example, soy protein decreases *Bacteroidetes* abundance, whereas fish or beef protein increases it [58]. Brown rice and egg white proteins increase gut amino acid-degrading enzyme activity, and egg white protein enriches bacteria associated with intestinal mucus degradation [59].

This likely contributes to inconsistent microbiota responses observed in human dietary interventions, reflecting dietary co-variables, heterogeneous protein sources, and strong interindividual variability. Low-protein plant-based diets are associated with a reduced abundance of gut UT-related genes [2], while CKD progression is accompanied by a decrease in the plant-to-animal carbohydrate-active enzyme (CAZyme) ratio, a proxy for carbohydrate versus animal protein fermentation capacity [3].

Dietary fiber interventions, mainly through increased plant-based diets, modify carbohydrate metabolism [60], induce enterotype shifts, inhibit microbial tryptophanase and decrease indole production [61], thereby reducing plasma UT levels in CKD [61,62] (Table 2). Short-chain fatty acids (SCFAs), the main end products of carbohydrate fermentation in the gut, maintain intestinal barrier integrity, modulate immune activation, reduce systemic inflammation and protect against kidney disease progression via GPR43 and GPR109A signaling [63,64,65]. Some studies reported that direct supplementation with SCFAs at different stages of CKD may reduce UT levels [66,67]. In CKD, SCFA production capacity is reduced and is associated with lower fecal butyrate levels [63,68], likely exacerbated by decreased fiber substrate availability. Under basal conditions and in in vitro models, however, differences in SCFA production are not always significant and further exploration of this pathway is required in CKD [9].

Plant-based diets are often associated with a risk of hyperkalemia in this population. However, this concern may be overstated, and several studies suggest that the association is likely modest. Plant foods have properties that may limit potassium retention, including alkalinizing effects, lower potassium bioavailability, and the role of dietary fiber in promoting colonic potassium excretion. These findings support patient-centered kidney diets that include plant-based foods rather than systematically restricting them [69]. Altogether, these findings indicate a progressive shift toward protein-oriented microbial metabolism during CKD progression, favoring UT production at the expense of carbohydrate fermentation and protective metabolites such as SCFAs, thereby reinforcing the rationale for plant-based low-protein diets (Table 2).

#### 3.1.3. Specific Amino Acid Composition of Proteins

Finally, beyond protein quantity, the amino acid composition of proteins is likely another critical determinant of the impact on the gut microbiota and UT production. In non-CKD conditions, studies have shown that restriction of one or a few specific amino acids, especially tryptophan, is sufficient to improve metabolic parameters so they are comparable to those observed with global protein restriction. This effect includes activation of fibroblast growth factor 21, a key endocrine sensor of amino acid imbalance that may confer important metabolic benefits [70].

Similarly, in CKD, not all amino acids exert equivalent effects within the gut–kidney axis. Lobel et al. demonstrated that increased intake of methionine and cysteine, sulfur-containing amino acids, improves kidney function in CKD mice [71]. Mechanistically, sulfide produced from bacterial metabolism of sulfur-containing amino acids regulates *Escherichia coli* indole, ammonia and urea production through inhibition of tryptophanase via S-sulfhydration, a post-translational modification of cysteine residues in this enzyme. Conversely, restriction of dietary sulfur-containing amino acids appears to exert protective effects against acute kidney injury in experimental models. However, in a randomized clinical trial (RCT), this type of specific protein restriction was not associated with any protective effects against acute kidney injury after cardiac surgery. Importantly, the study demonstrated that this type of selective dietary approach was feasible in clinical settings [72]. In 5/6 nephrectomized rats, diets containing 10% branched-chain amino acids increased renal fibrosis compared with diets enriched with aromatic amino acids (i.e., tryptophan, tyrosine, and phenylalanine), which instead stimulated renal plasma flow and, to a lesser extent, glomerular filtration rate [73]. Consistently, preliminary data suggest that a reduction in specific aromatic amino acids in CKD mice decreases PCS and IS production and confers nephroprotection at levels comparable to those observed with global protein restriction [74].

However, the impact of amino acid composition on CKD progression and UT generation has not yet been explored in humans and currently remains more of a conceptual framework than a practical dietary recommendation. Nevertheless, it opens perspectives for future protein design strategies tailored to CKD [75].

### 3.2. Lipids

Because most UTs originate from amino acid metabolism, the impact of dietary lipids on UT production and gut microbiota modulation has been far less and even largely unstudied in CKD. Therefore, the causal pathways linking lipid intake to UT generation in CKD remain largely hypothetical, as most studies have been conducted in the general population.

Yet lipids account for more than 30% of global energy intake in Western diets, and several animal and human studies report long-term associations between high-fat diets and kidney alterations [76]. Qualitative aspects of dietary fat intake, including lipid type, source, and supplementation, therefore warrant specific attention.

In the general population, RCTs support lipid-mediated microbiota modulation. In a six-month controlled-feeding study of 217 healthy adults, lower-fat diets increased microbial alpha diversity and *Blautia* and *Faecalibacterium* abundance, whereas higher-fat diets increased *Alistipes* and *Bacteroides* while reducing *Faecalibacterium* [77]. *Faecalibacterium* is known as a butyrate-producing species associated with nephroprotection [63]. Total SCFA concentrations were lower in the higher-fat group, and microbial by-products such as p-cresol and indole decreased in the lower-fat group. Consistently, low saturated fat intake is associated with greater microbial diversity [78]. Rodent studies [79] and several clinical cohorts [80] reported increased plasma TMAO with high-fat diets, although results remain inconsistent, partly due to unreported phospholipid intake [81]. Saturated fats promote pro-inflammatory microbial profiles, whereas polyunsaturated fats favor beneficial taxa, highlighting the complexity of lipid–microbiota interactions [82].

Interest in lipid supplementation has increased also due to its ability to modify polyunsaturated fatty acid (PUFA) intake. PUFAs include plant-derived alpha-linolenic acid and marine-derived eicosapentaenoic acid (EPA), docosapentaenoic acid (DPA), and docosahexaenoic acid (DHA). In a randomized crossover pilot study, 30-day supplementation with soluble corn fiber plus fish oil altered gut microbial composition and increased butyrate-producing taxa compared with a Mediterranean diet plus corn oil [83]. Omega-3 fatty acids also modify gut microbiota, with some CV benefits mediated through microbial fermentation by-products [84], while milk polar lipids improve cardiometabolic markers without altering major bacterial phyla [85].

Current KDIGO guidelines [22] do not recommend routine long-chain omega-3 supplementation beyond triglyceride lowering. However, this position may warrant reassessment following the PISCES trial, which for the first time demonstrated that a nutritional intervention strikingly reduced CV mortality in hemodialysis patients [86]. This trial should be interpreted with caution, as early trials in non-CKD populations using low-dose omega-3 supplementation yielded mixed results. The GISSI Heart Failure trial suggested modest benefits in a population with limited background cardiovascular therapy, whereas later trials in statin-treated populations failed to confirm these effects, as exemplified by ASCEND [87]. Given CKD-specific dysbiosis characterized by excess pro-inflammatory UTs, omega-3–microbiota interactions, their potential impact on UTs, and their mechanisms of action warrant dedicated investigation [2,88].

### 3.3. Sugar and Sweet Foods

Sugar represents the third major macronutrient class and can influence gut microbiota composition and metabolic homeostasis. Experimental data suggest that high sugar intake may promote the expansion of *Erysipelotrichaceae* and reduce beneficial bacteria, potentially leading to the loss of immune-regulating microbes involved in the prevention of metabolic syndrome [89]. In humans, observational data indicate that lower consumption of sugar-sweetened or artificially sweetened beverages is associated with a reduced risk of CKD [90]. In the UK Biobank, higher free sugar intake was associated with increased CKD risk, whereas non-free sugars appeared as protective. These associations were modified by gut microbial abundance and were strongest in individuals with genetically predicted high microbial abundance, highlighting potential host–microbiota interactions in mediating dietary effects [91].

Artificial sweeteners include non-nutritive sweeteners (NNSs) such as aspartame, sucralose, saccharin and acesulfame K, as well as low-calorie polyols including xylitol, erythritol and sorbitol. The first studies are very convincing about the role of NNSs in inducing gut microbiota dysbiosis. In rodents, exposure to sucralose and maltodextrin reduced the abundance of *Bifidobacterium*, *Lactobacillus* and Bacteroides [92,93]. In a study of 172 NNS consumers, 16S rRNA profiling revealed enrichment of *Enterobacteriaceae*, *Deltaproteobacteria* and *Actinobacteria*, together with higher fasting glucose, adiposity and glycated hemoglobin levels [92].

As highlighted in recent systematic reviews, animal studies consistently report that NNS exposure reduces beneficial taxa such as *Bifidobacterium* and *Lactobacillus* while increasing potentially harmful bacteria including *Clostridium difficile* and *Escherichia coli.* Alterations in SCFA production have also been described. However, human data remain limited and often inconsistent, likely reflecting differences in study design, dose, duration and sweetener type [94]. Overall, although artificial sweeteners may reduce caloric intake and glycemic load, accumulating evidence suggests potential effects on gut microbiota and metabolic pathways. Importantly, no dedicated studies have specifically examined these effects in CKD, and even fewer data are available regarding their impact on UTs.

### 3.4. Micronutrients

#### 3.4.1. Polyphenols and Other Phytochemicals

Approximately 8000 polyphenols have been identified. Polyphenols are plant-derived bioactive compounds characterized by multiple phenolic units, including flavonoids (quercetin, catechins, and anthocyanins), phenolic acids (caffeic and ferulic acids), curcuminoids (curcumin), stilbenes (resveratrol), and lignans. Other phytochemicals are also provided by plants, such as isothiocyanates (e.g., sulforaphane from broccoli and other cruciferous vegetables). Polyphenols interact bidirectionally with the gut microbiota: they promote beneficial taxa and inhibit pathogenic bacteria, while microbial metabolism converts polyphenols into bioactive derivatives with enhanced bioavailability [10]. Polyphenol-rich foods (tea, berries, grapes, and pomegranates) exert prebiotic-like effects by enriching *Lactobacillus* and *Bifidobacterium* and reducing potentially harmful genera such as *Clostridium*, with downstream effects on host metabolism and inflammation.

Most interventional evidence originates from populations with metabolic diseases. Broccoli sprout extract, rich in sulforaphane, has been shown to improve glucose homeostasis, and emerging data suggest that interindividual response may be partly related to gut microbial features, including *Bacteroides*-related metabolic pathways involved in sulforaphane activation [95]. Curcumin supplementation in prediabetes modestly enriched beneficial taxa including *Bacteroidota*, *Bacteroides*, and *Roseburia* while maintaining glucose homeostasis [96]. A meta-analysis of 13 trials (n = 670) reported reduced metabolic endotoxemia, increased antioxidant activity, and enhanced SCFA production following polyphenol supplementation, although effects on inflammation and body weight were inconsistent [97].

In CKD, evidence remains limited but promising. A systematic review of 32 studies showed that curcumin or turmeric reduced C-reactive protein, particularly in dialysis patients [98]. In stage 3 CKD with diabetes, six weeks of resveratrol improved endothelial function [99]. Small clinical studies also suggest that curcumin and other polyphenols may reduce UT production, possibly via gut microbiota modulation (Table 2). Overall, polyphenols beneficially modulate gut microbiota and microbial metabolites, with potential metabolic and anti-inflammatory effects, but dedicated CKD studies are still needed to clarify mechanisms and long-term renal outcomes.

#### 3.4.2. Alcohol

Alcohol consumption remains a relevant issue in patients with CKD, with approximately 20–36% of patients reporting occasional or daily intake and nearly 10% classified as heavy drinkers [100]. Chronic alcohol consumption induces gut dysbiosis, increases intestinal permeability, and reduces anti-inflammatory, butyrate-producing bacteria [101]. Alcohol dysbiosis is characterized by reduced *Bacteroidetes* and *Ruminococcaceae*, increased *Proteobacteria* and *Actinobacteria*, and endotoxemia; importantly, several of these alterations partially reverse with alcohol abstinence [101].

Alcohol-induced microbiota changes appear directly related to alcohol intake and may contribute to a vicious cycle of consumption. Fecal microbiota transplantation studies support this concept, showing that transplantation of feces from non-alcohol-consuming humans was associated with reduced alcohol intake and preference in murine models [102]. In addition, patients with severe alcoholic hepatitis exhibit reduced levels of bacterial tryptophan-derived aryl hydrocarbon receptor (AhR) agonists, further highlighting microbiota-mediated metabolic alterations [103].

In CKD, integrative supervised multi-omics modeling comparing moderate and severe disease identified alcohol consumption as one factor influencing CKD-associated gut microbiota dysbiosis, suggesting that alcohol-related microbial alterations may further amplify uremic toxicity and UT production in advanced CKD [2]. However, there are substantial inconsistencies between experimental and clinical studies on alcohol consumption and kidney damage, and the relationship between alcohol and kidney outcomes remains unclear [104]. This specific relationship therefore warrants further investigation in dedicated experimental studies.

#### 3.4.3. Other Supplementation: Biotics, Iron, Minerals and Vitamins

Beyond dietary composition itself, many additional compounds of diverse origin are frequently grouped under the broad term “nutritional supplements”. These include vitamins, ions and metals such as iron, and biotics (pre-, pro-, syn-, and postbiotics). This complexity makes it particularly difficult to disentangle the specific effects of supplementation on gut microbiota. Importantly, many of these compounds are not regulated by health authorities to the same standards as medications, and their composition is often poorly defined, which has led to several cases of intoxication and even death, as recently reported with extracts from red yeast rice [105].

Nevertheless, some supplements, particularly pre- and probiotics, may represent valuable tools for microbiota modulation and a reduction in UTs [8,106], although a recent meta-analysis did not clearly support the efficacy of this strategy, likely reflecting empirical selection of strains and formulations [107]. A step-by-step approach, from in silico to in vitro and in vivo models, should be adopted for the selection of biotics to enable more effective strategies for reducing UT production, as recently proposed by Beau et al. [9]. They have shown encouraging effects on microbiota composition and UT modulation in CKD [9]. Also more targeted approaches may be required: for example, probiotics producing SCFAs, such as *Lactobacillus casei* Zhang or *Faecalibacterium prausnitzii*, appear promising in CKD contexts [63,64]. Probiotics may also play a major role [108]. For example, short-chain fatty acids (SCFAs), particularly butyrate, may contribute to shaping gut microbiota composition, modulating intestinal permeability [65], and exerting anti-inflammatory effects [109].

Biotics supplementation can also be combined with a LPD (Table 2). For example, a low-salt LPD supplemented with 10 g of inulin (a dietary fiber) in 45 CKD patients significantly reduced serum IS and PCS levels more than an LPD diet alone [35].

Regarding vitamins, no interventional studies have addressed their interactions with the gut microbiota and UTs in CKD. Preliminary data suggest that *Bacteroides*, a key vitamin K2-producing genus, is reduced in patients with inadequate vitamin K status [35], highlighting a potential link between microbial alterations and micronutrient deficiencies.

For mineral supplementation, higher magnesium [110] and zinc intake [111] has been associated with better CKD outcomes in observational cohorts. Given their potential impact on gut microbiota composition [112], both the quantity of intake and their effects on UT production should be more specifically considered.

Altogether, these observations underscore the need for rigorously designed, transparent, and regulatory-authorized intervention studies in CKD to define safe, mechanism-based supplementation strategies and avoid unintended toxicity while maximizing microbiota-mediated benefits.

### 3.5. Dietary Patterns

#### 3.5.1. Food Structure

More recently, a shift toward a holistic approach to dietary patterns has emerged, moving beyond isolated nutrients toward integrated nutritional strategies. The Mediterranean diet is defined by a high consumption of fruits, vegetables, whole grains, legumes, nuts, and olive oil, with moderate intake of fish, poultry, and dairy products, and a limited intake of red meat and sweets. The Dietary Approaches to Stop Hypertension (DASH) diet is characterized by a dietary pattern rich in fruits and vegetables. Both these diets have demonstrated benefits through improved gut microbiota diversity, enhanced microbial balance, and reduced inflammation and oxidative stress in metabolic diseases and CV diseases [113], although findings are not entirely consistent [114]. The focus is increasingly on dietary patterns (holistic approach) that promote overall health and disease prevention in CKD rather than specific restriction of individual nutrients (reductionist approach).

Over the past decade, observational studies in CKD populations have consistently shown that adherence to dietary indices such as the Healthy Eating Index, Mediterranean diet, DASH, and planetary health diet, or a higher intake of healthy plant-based foods is associated with improved outcomes across diverse populations [115,116,117,118]. However, RCTs remain limited, making causal inference difficult. Furthermore, it remains unclear to what extent these benefits are mediated by gut microbiota modulation and microbial metabolite production.

Wang et al., in the largest observational study including over 250 hemodialysis patients, found no significant association between dietary patterns, fecal microbiota composition, and UT levels [20]. Importantly, this cross-sectional study involved late-stage CKD patients and was not designed to evaluate dietary modification longitudinally. In contrast, Laiola et al. reported that plant-based LPDs were associated with a reduced abundance of bacterial genes involved in UT production [2].

Table 2 summarizes clinical trials assessing dietary patterns in relation to gut microbiota and UT production. These studies are generally limited by small sample sizes, heterogeneous CKD stages, short-term interventions, incomplete integration of the exposome and treatment-related confounders, and the absence of hard outcomes such as eGFR slope. A recent randomized crossover trial in 25 adults with stage 3–4 CKD showed that increasing plant diversity (≥30 plant foods/week) reduced symptom burden and shifted the microbiome toward more beneficial metabolite production, particularly in individuals with advanced CKD and higher baseline UT levels [33]. Other dietary strategies, including the Mediterranean diet and the New Nordic Renal Diet, have also shown encouraging reductions in UTs and microbiota modulation [36,40].

Multifunctional nutritional strategies combining several bioactive compounds may exert synergistic effects on inflammation, gut microbiota modulation, and cardiometabolic profiles. A two-month intervention integrating multiple bioactive components (in this study, cereal products enriched with polyphenols, fibers, slowly digestible starch, and omega-3 fatty acids) improved intestinal inflammation and induced measurable microbiota changes [119], supporting the potential of integrated dietary approaches in CKD.

Finally, the Mediterranean diet, in particular, should be considered not only as a dietary pattern but as a broader lifestyle approach. It follows long-standing principles of food production, emphasizing respect for natural ingredients and favoring simple cooking methods. Fresh, seasonal, and locally sourced plant-based foods form the core of this diet, which could strongly influence the production of UTs, although this has not yet been directly assessed.

#### 3.5.2. Food Processing

Beyond overall dietary patterns, the degree of food processing also appears to be a critical determinant of health outcomes. Higher consumption of ultra-processed foods (UPFs) is associated with an increased risk of developing CKD, although it remains unclear whether this reflects poor overall dietary quality, the cumulative effects of food additives, or displacement of healthier dietary patterns [120]. Food additives used for coloring, flavoring, texturizing, and preservation have been shown to increase intestinal permeability [121], potentially worsening gut microbial composition and enhancing UT production. In the French NutriNet-Santé cohort, which provides detailed characterization of food processing, high adherence to a nutritionally healthy, minimally processed plant-based diet was associated with a 44% lower incidence of coronary heart disease and a 32% reduction in CV risk, while higher consumption of processed meat was associated with increased IS levels, even in healthy populations [122,123]. Similar approaches are needed in CKD to determine whether these effects are mediated by gut microbiota and UTs.

## 4. Gut Transit Time and Constipation as Modulators of Uremic Toxin Generation

Colonic transit time is closely related to gut microbiota-derived metabolism and mucosal turnover [124] and has been identified as a risk factor for CKD progression [125]. In CKD, reduced intake of fruits and vegetables due to concerns about hyperkalemia often leads to low fiber consumption, thereby increasing the prevalence of constipation. Prolonged intestinal transit may further exacerbate UT levels by increasing substrate availability, enhancing proteolytic fermentation, and prolonging intestinal contact time, thereby facilitating UT absorption.

Accordingly, modulation of bowel transit through dietary fiber or pharmacological agents has emerged as a potential strategy to reduce UT burden. Laxatives such as lubiprostone and linaclotide have been associated with reduced circulating UT levels in both experimental models and human uremic conditions [126,127], likely by accelerating transit and shifting microbial metabolism toward saccharolytic pathways.

Consistent with this concept, a recent study identified intestinal transit time as a primary determinant of gut microbial variability in CKD and demonstrated increased proteolytic activity with advancing disease stage [3]. These findings highlight constipation and delayed transit as modifiable contributors to dysbiosis and UT generation, supporting the integration of bowel habit management into nutritional strategies for CKD.

## 5. Meal Timing and Circadian Influences on Gut Microbiota and Uremic Toxin Production

Emerging evidence suggests that meal timing may influence kidney and metabolic health. For example, earlier intake of unsaturated fatty acids was associated with improved insulin sensitivity, likely mediated through modulation of the gut microbiota [128]. In CKD, analyses from the NHANES cohort reported significant associations between tea consumption timing and kidney function, with the strongest associations observed during the morning period [129].

Renal physiology is tightly regulated by circadian rhythms and may respond differently depending on the timing of food intake and UT production. At the molecular level, AhR exhibits circadian expression and interacts with core clock genes, influencing metabolic rhythms and glucose homeostasis. However, its regulation in CKD and the role of UTs and gut microbiota in circadian disruption remain poorly understood [130,131].

Intermittent fasting (IF) has emerged as a common chrononutritional strategy. Beyond caloric restriction, IF improves metabolic health through modulation of gut microbiota, bile acid signaling, and inflammatory pathways [132]. Experimental data suggest that IF may limit progression from acute kidney injury to CKD [133]. IF reshapes gut microbial composition, increasing *Lactobacillus* abundance and improving metabolic parameters [134,135].

Small clinical studies in CKD indicate short-term feasibility under medical supervision, with modest weight loss and improvements in eGFR, although long-term renal safety remains uncertain [136]. Robust clinical trials in CKD are still lacking, and careful evaluation of long-term renal outcomes is required before clinical implementation.

## 6. Can Uremic Toxin Profiling and Gut Microbiota Composition Be Used to Design a Personalized Diet?

A major challenge in CKD is individualizing nutrition and developing targeted dietary strategies tailored to patients’ specific needs rather than applying uniform recommendations. Interindividual variability in dietary responses appears partly microbiota-dependent. Cotillard et al. showed that overweight individuals with higher gut microbiota richness exhibited stronger anti-inflammatory responses to a low-calorie diet [137], while Kovatcheva-Datchary et al. reported improved glucose metabolism after a barley-rich diet in individuals with higher *Prevotella copri* abundance [138]. However, direct assessment of gut microbiota remains challenging due to limited biosample availability, high interindividual variability influenced by factors such as intestinal transit, and the lack of large-scale sequencing studies identifying robust microbial markers of CKD progression [2,3]. Circulating metabolites, including UTs, may therefore represent a more practical surrogate of microbiome-driven heterogeneity and a potential readout for precision nutrition [139] with artificial intelligence offering tools to integrate these multidimensional datasets.

The feasibility of personalized nutrition has been demonstrated in metabolic diseases, where individualized dietary recommendations integrating host, environmental, and microbial variables resulted in greater improvements in postprandial glycemic control than standard dietary advice [6]. Similarly, multidimensional personalized diets incorporating glycemic response, blood biomarkers, dietary habits, anthropometrics, physical activity, and gut microbiota yielded superior metabolic improvements [140,141]. Personalizing a diet also requires consideration of food preferences and acceptability. The kidney–gut–brain axis may contribute [142], as UT accumulation can impair blood–brain barrier integrity and cognition, potentially affecting appetite regulation [143]. Experimental data further suggest that the gut microbiota can influence macronutrient preference and feeding behavior [144], although clinical evidence in CKD remains scarce. Future studies should determine whether biomarker-guided nutritional strategies improve gut microbiota composition, UT profiles, and ultimately CKD outcomes.

## 7. Clinical Challenge

Adherence to restrictive dietary patterns remains a major challenge in CKD populations, reflecting both patient-level constraints and structural barriers. In randomized trials of low-protein diets, fewer than 20% of patients achieved full adherence [145], highlighting the substantial demands in terms of time, sustained dietary counseling, and impact on daily life. This should be interpreted in the broader context of chronic disease management, where adherence to long-term pharmacological treatments is also suboptimal, with approximately 50% of patients remaining adherent to antihypertensive therapy at one year [146].

However, when dietary interventions are effectively implemented, their clinical impact can be meaningful. Evidence suggests that adherence to low-protein or plant-based dietary strategies may reduce healthcare costs [147], compared with usual care.

These observations underscore the need for structured, multidisciplinary education programs integrating dietitians, clinicians, and behavioral support. Interventions should also extend beyond specialized nephrology care, incorporating public health strategies to improve nutritional literacy, accessibility, and long-term adherence to kidney-friendly dietary patterns.

## 8. Conclusions

**Figure 1:**
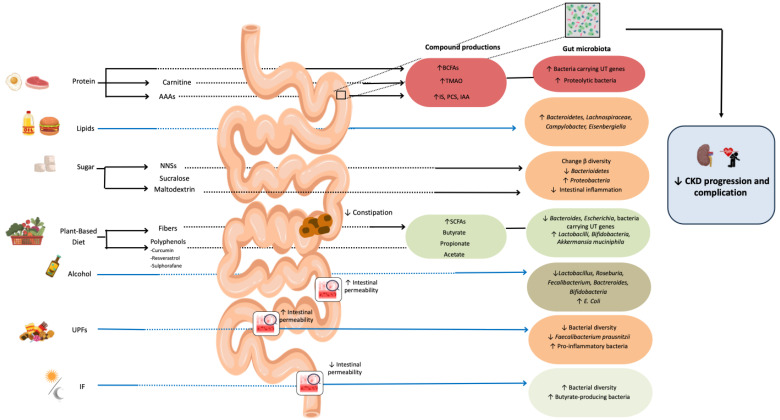
Impact of diet on gut microbiota structure and uremic toxin production. Abbreviations: AAAs: aromatic amino acids; BCFAs: branched-chain fatty acids; IAA: indole-3-acetic acid; IF: intermittent fasting; IS: indoxyl sulfate; NNSs: non-nutritive sweeteners; PCS: p-cresyl sulfate; SCFAs: short-chain fatty acids; TMAO: trimethylamine N-oxide; UPFs: ultra-processed foods; UTs: uremic toxins. The blue arrow underlines that the evidence is mainly based on the general population.

Diet is now recognized as a key modulator of gut microbiota activity and UT production, as summarized in Figure 1. Understanding how specific dietary patterns shape the UT–gut–kidney axis is essential to move toward precision nutrition in CKD, enabling personalized interventions based on individual risk of progression and CV complications.

However, it is important to emphasize that current evidence remains largely associative. While numerous studies have reported links between UT levels and CKD severity, far fewer have demonstrated a causal relationship with CKD progression. Moreover, despite promising experimental data, no clinical study to date has conclusively shown that reducing UTs through nutritional interventions translates into a slowing of CKD progression or a reduction in hard clinical outcomes. This highlights the need to clearly distinguish between associations with CKD and true effects on disease progression. In addition, some studies have reported limited or inconsistent effects of dietary interventions on UT levels or clinical outcomes, underscoring the complexity of diet–microbiota interactions and the influence of major confounders. These findings call for a more balanced interpretation of the field.

Finally, major knowledge gaps remain regarding how the food matrix, nutrient composition, and microbial metabolism interact to drive UT generation. Robust biomarkers capturing these diet–microbiota–toxin relationships are urgently needed to guide clinical decision-making. To date, no study has defined which biomarkers should be used, how many are needed, or how they should be implemented. Other important questions remain, such as cost, reproducibility, and turnaround time. Large cohort studies using artificial intelligence-based approaches, as well as interventional studies, are needed to demonstrate their clinical value in patients with CKD. In addition, factors such as environmental exposures, food processing and cooking methods, and meal timing are likely to further modulate this axis and must be integrated into future research. Most current studies mainly focus on UT metabolites without analyzing the full metabolome, even though beneficial microbial metabolites that should be promoted may also play an important role. Advancing this field will require interdisciplinary approaches combining microbiome science, metabolomics, nutrition, and systems biology. Ultimately, elucidating the diet–microbiota–UT pathway offers a promising avenue to refine personalized nutrition strategies and improve kidney and CV outcomes in CKD.

## Data Availability

Data sharing is not applicable.

## Abbreviations

Glossary: 16S rRNA gene sequencing: A method based on sequencing the 16S ribosomal RNA gene to identify and classify bacteria, mainly providing taxonomic information with limited functional insight. Archaeome: The community of archaea present in a given environment. Metagenomics: The analysis of the total genetic material from all microorganisms in a sample, allowing for identification of both microbial taxa and their functional potential. Mycobiome: The community of fungi inhabiting a specific environment. Taxonomic: Relating to the classification and identification of organisms into groups such as species, genus, or family. Virome: The collection of viruses present in a given environment.
